# New insights in respiratory impedance in young children after repair of congenital diaphragmatic hernia: a cross-sectional study

**DOI:** 10.1186/s13052-019-0670-6

**Published:** 2019-07-15

**Authors:** Giuliana Ferrante, Giovanna Cilluffo, Maria Rita Di Pace, Giovanni Corsello, Enrico Lombardi, Raffaele L. Dellacà, Velia Malizia, Stefania La Grutta

**Affiliations:** 1 Department of Health Promotion Sciences Maternal and Infant Care, Internal Medicine and Medical Specialities, University of Palermo, via del Vespro 129, 90127 Palermo, Italy; 2 National Research Council of Italy, Institute of Biomedicine and Molecular Immunology, via Ugo La Malfa 153, 90146 Palermo, Italy; 3 Pediatric Pulmonary Unit, “Anna Meyer” Pediatric University-Hospital, Viale G. Pieraccini 24, 50139 Florence, Italy; 4 Dipartimento di Elettronica, Informazione e Bioingegneria, Politecnico di Milano University, Milano, Italy

**Keywords:** Children, Congenital diaphragmatic hernia, Forced oscillation technique, Lung function, Respiratory impedance

## Abstract

Lung function impairment is common in Congenital Diaphragmatic Hernia (CDH) survivors. The aim of this study was to evaluate, in children who underwent CDH surgical repair, mid and long-term consequences on respiratory impedance, investigating the impact of CDH on both resistance and reactance parameters, as well as bronchodilator response.

Forced Oscillation Technique (FOT) parameters were collected from 12 patients (2–11 years). Resistance and reactance values at 8 Hz (Rrs8, Xrs8) and the area under the reactance curve (AX) were measured pre and post-salbutamol. Quantitative variables were compared using Mann-Whitney U test. Differences of categorical variables were evaluated using Fisher exact test. Statistically significant differences between measured and predicted values for Rrs8 (*p* = 0.04), Xrs8 (*p* = 0.02) and AX (*p* = 0.01) were found. When stratifying for age, significant difference between measured and predicted values was observed only in children < 5 years (*n* = 6) (Rrs8 *p* = 0.03, Xrs8 *p* = 0.001, AX *p* = 0.007). With respect to children 5 years (*n* = 6), the younger ones showed higher z-scores in Rrs8 (*p* = 0.015), Xrs8 (*p* = 0.002) and AX (*p* = 0.002) values. Since the z-score difference was greater than 0.5, it was considered a difference clinically relevant. No differences in bronchodilator response were recorded.

In children with CDH an impairment of respiratory impedance measured by FOT is observed only in children aged less than 5 years.

To the Editor,

Congenital diaphragmatic hernia (CDH) is a rare malformation of the lung, occurring in 1–5/10,000 births (https://www.orpha.net), due to a defect in the diaphragm that allows abdominal organs to move into the chest cavity. Variable degrees of impairment of lung function in CDH survivors have been reported throughout childhood [1], though compensatory lung growth is expected to be completed by the age of 6 years [2].

The Forced Oscillation Technique (FOT) provides detailed characterization of the resistance (Rrs) and reactance (Xrs) of the respiratory system over a wide range of frequencies, which respectively reflect the frictional losses and the energy storage (elastic and inertial) properties of the respiratory system. Moreover, FOT is able to detect reversible airway obstruction in children. The resistance measured contains contributions from both the lung and chest wall, whereas reactance yields information about the elastic properties of the lung. In general, lower frequency data reflect the peripheral regions of the lung, while higher frequency data are most representative of the central airways [3]. To our knowledge, only one study evaluated lung function by means of FOT in children surviving after CDH repair, finding that resistance at 8 Hz (Rrs8) was increased in 60% of the patients [4]. However, this study did not explore changes to respiratory reactance, which provides much more sensitive information on lung periphery compared to Rrs8, nor the bronchodilator response.

The aim of this study was to evaluate, in children who underwent CDH surgical repair, mid and long-term consequences on respiratory impedance, investigating the impact of CDH on both resistance and reactance parameters, as well as bronchodilator response.

From 2003 to 2011, a total of 16 children born with CDH referred to the Pediatric Surgical Unit of the University of Palermo, Italy, underwent surgical repair at birth. As a part of their multidisciplinary follow-up, CDH children aged 2–11 years were enrolled at the Pediatric Pulmonology Outpatient Clinic of the IBIM National Research Council (CNR) in Palermo, Italy. Twelve (75%) agreed to participate in the study. Mental retardation (*n* = 1), emigration (*n* = 1) and absence of follow-up (*n* = 2) were causes of exclusion from the study. Clinical characteristics were comparable between participants and those not included (Table 1).

**Table 1:** Comparison of clinical characteristics of included and excluded participants

	Included	Excluded	*p*-value*
	*n* = 12	*n* = 4	
Inborn delivery	3 (27%)	4 (100%)	0.056
1-min Apgar score	6 (4.5–6.5)	5 (5.0–5.5)	0.749
5-min Apgar score	8 (8.0–9.25)	9 (8.5–9.0)	0.540
Duration of artificial ventilation, days	6 (6–13)	25 (16.0–31.25)	0.160
Total hospitalization length, days	24 (17.75–35.25)	60 (60–60)	0.098
Surfactant administration	3 (30%)	1 (25%)	1.000
Type of defect size^a^			0.784
B	6 (50%)	3 (75%)	
C	5 (41%)	2 (25%)	
D	1 (8%)	0 (0%)	
Surgical repair			0.379
Primary closure	9 (75%)	2 (50%)	
Patch reconstruction	3 (25%)	2 (50%)	

Data are presented as n (%) or median (25th–75th percentiles). *Fisher information test or Mann-Whitney U test; ^a^Congenital Diaphragmatic Hernia Study Group. Congenital diaphragmatic hernia: defect size correlates with developmental defect. J Pediatr Surg. 2013;48:1177e82

The study was performed in accordance with the Declaration of Helsinki and Good Clinical Practice guidelines; it was approved by the local ethics committee and registered on the central registration system (NCT02466451).

Inclusion criteria were the following: (i) history of CDH; (ii) surgical repair at birth; (iii) written informed consent; (iv) ability to undergo FOT. Exclusion criteria were the following: (i) major congenital cardiac, pulmonary and neurological anomalies [5]; (ii) immunological and/or metabolic disease; (iii) upper or lower respiratory tract infections in the last 2 weeks; (iv) therapies with antibiotics and/or corticosteroids in the last 2 weeks.

Detailed medical information on prenatal CDH ultrasound diagnosis, mode of delivery, Apgar score, birth weight, duration of artificial ventilation and hospitalization, surfactant administration, CDH defect size, surgical repair, personal history of respiratory infections and gastroesophageal symptoms, current passive smoking exposure, was obtained by trained medical investigators.

Height and weight were measured using a stadiometer (Wunder HR1, Italy) and an electronic weighing scale (Seca, Hamburg, Germany). Respiratory impedance was measured using the Quark i2m® Forced Oscillation Measurement system (Cosmed, Italy) based on a pseudo-random noise signal between 4 and 48 Hz, according to the ATS/ERS recommendations [6]. Subjects were seated with the head in a neutral position, wearing a nose clip and breathing into a mouthpiece connected to the device; the cheeks and the floor of the mouth were gently supported by one of the investigators. Only measurements with a minimal coherence function of 95% and a coefficient of variation < 15% were considered valid. The mean value of three valid measurements was retained [3]. Bronchodilator response (BD) was evaluated repeating measurements 15 min after inhalation of salbutamol 200 μg [3] administered via a metered dose inhaler through a plastic spacer device (Aerochamber Plus®, Trudell Medical International, Canada).

Considering a sample size of 12 subjects, a power of about 79% in detecting a standardized effect size of 0.8 through a proportion test with a 5% family-wise error rate was obtained. Quantitative variables were compared using the Mann-Whitney U test. Differences of categorical variables were evaluated using the Fisher exact test. Z-scores of Rrs8, Xrs8 and the area under the reactance curve (AX) were computed according to equations established by Calogero et al. [3]. Analyses were performed using R (3.4.1) statistical software; a *p*-value < 0.05 was considered significant and a difference of 0.5 in Z-scores was considered clinical relevant [7] .

CDH was diagnosed prenatally by ultrasound in four patients with type “C” and in one with type “D” defect size [8]; no mentions of observed/expected lung volumes measurements were available. All children (41.67% males, mean age 5.01 ± 2.69 years) were full-term born (mean gestational age 39.7 ± 0.96 weeks) by cesarean section. Mean birth weight was 2847.5 ± 571.84 g, and 17% were small for gestational age. At the time of the examination, 25% suffered from lower respiratory tract infections in the first 2 years of life and 33% had doctor diagnosed gastroesophageal reflux, and 42% were currently exposed to passive smoking.

All children successfully underwent FOT measurement. The difference in Rrs8 (Fig. 1, panel A), Xrs8 (panel B) and AX (panel C) absolute values from their predicted ones in relation to age were greatest in younger children and reduced with age for the older patients. Differences between measured and predicted values were significant for Rrs8 (*p* = 0.04), Xrs8 (*p* = 0.02) and AX (*p* = 0.01).

**Fig. 1:**
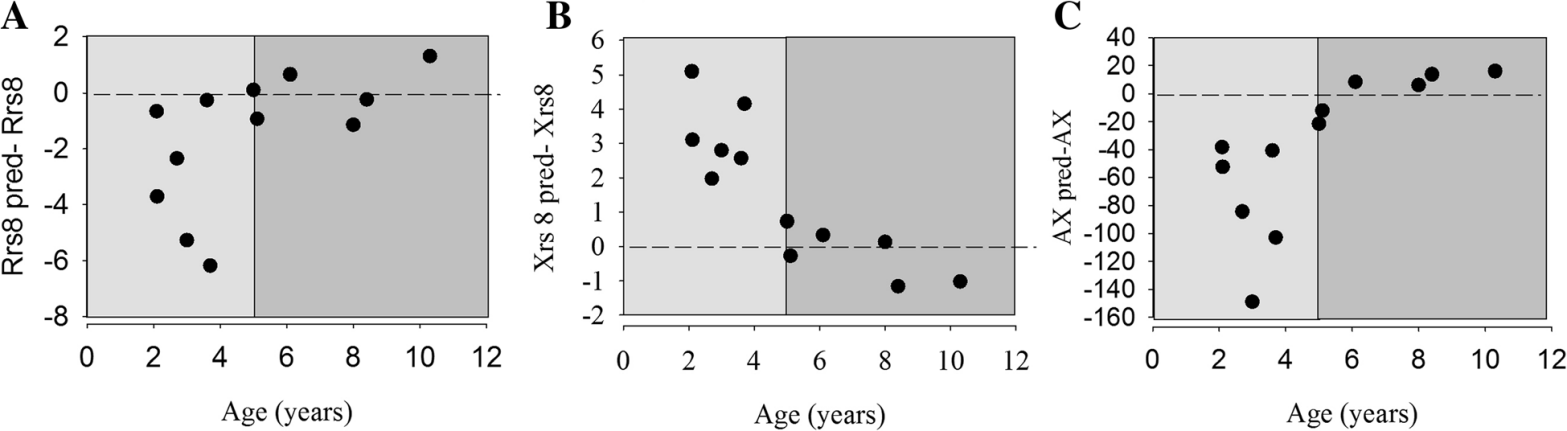
Distribution of differences from predicted values of Rrs8 (panel **a**), Xrs8 (panel **b**), AX (panel **c**). Points in light grey and in dark grey sides of the figure indicate differences from predicted values of Rrs8, Xrs8, AX in children aged < 5 years and in children aged ≥5 years, respectively

In Table 2, comparisons between CDH children aged < 5 years and ≥ 5 years are reported. No significant differences between study groups were found in clinical characteristics. Younger children had significant higher z-scores and higher %predicted FOT parameters.

**Table 2:** Patients characteristics, initial management and respiratory impedance by age

	All	< 5 years	≥5 years	*p*-value*
	*n* = 12	*n* = 6	*n* = 6	
Inborn delivery	3 (27%)	3 (42.86%)	0 (0%)	0.181
1-min Apgar score	6 (4.5–6.5)	6 (5–7)	6 (3–6)	0.309
5-min Apgar score	8 (8.0–8.0)	8 (8.0–8.75)	7.5 (6.5–8.0)	0.072
Duration of artificial ventilation, days	6 (6–13)	13 (6–15)	5 (4–7)	0.104
Total hospitalization length, days	24 (17.75–35.25)	24 (17.25–52.50)	24 (19.25–30.25)	0.872
Surfactant administration	3 (30%)	3 (50%)	0 (0%)	0.181
Type of defect size^a^				0.455
B	6 (50%)	2 (33%)	4 (66.67%)	
C	5 (41%)	2 (33%)	3 (50%)	
D	1 (8%)	1 (16.67%)	0 (0%)	
Surgical repair				1.000
Primary closure	9 (75%)	5 (83.33%)	4 (66.67%)	
Patch reconstruction	3 (25%)	1 (16.67%)	2 (33.33%)	
FOT				
Rrs8	9.33 (7.41–12.66)	12.73 (10.59–14.75)	7.26 (6.78–8.20)	
z-score	0.25 (−0.03–1.19)	1.43 (0.48–1.94)	−0.04 (0.12 - -0.31)	**0.015**
% predicted	8.95 (1.90–27.34)	31.74 (11.08–49.09)	1.2 (−6.28–8.99)	**0.041**
Relative change (% of baseline)	−20.83 (−29.78- -4.46)	−20.83 (−25.70- -16.91)	−14.54 (− 31.31 - -2.9)	0.930
Z-score change	−0.46 (−2.65–3.74)	−0.46 (− 0.98 - -0.45)	−0.06 (−4.62–13.79)	0.662
Xrs8	−4.39 (−5.98- -2.37)	−6.01 (− 6.80- -5.58)	−2.17 (− 2.91- -1.21)	
z-score	−1.36 (− 2.87–0.31)	−3 (− 3.85 - -2.61)	0.33 (− 0.19–0.94)	**0.002**
% predicted	1.35 (− 2.87 - -0.03)	2.95 (− 3.89- -2.62)	−0.07 (− 0.28–0.83)	**0.002**
Relative change (% of baseline)	31.86 (− 54.25 - -10.62)	33.59 (− 34.98 - -31.85)	17.85 (− 65.75- -0.40)	0.536
Z-score change	− 0.55 (− 0.92–0.16)	− 0.62 (− 0.67 - -0.55)	−0.22 (− 2.19–0.20)	0.536
AX	68.04 (32.61–101.37)	111.49 (82.41–139.61)	30.46 (12.59–45.77)	
z-score	1.44 (0.32–2.68)	2.86 (2.03–3.75)	0.04 (− 1.10–0.63)	**0.002**
% predicted	97.62 (18.0–145.79)	156.43 (112.58–239.54)	3.77 (− 53.61–52.06)	**0.004**
Relative change (% of baseline)	−32.25 (− 65.8- -10.24)	−32.93 (− 61.74 - -32.25)	− 10.24 (− 72.18–2.42)	0.428
Z-score change	−0.48 (− 1.15- -0.25)	− 0.48 (− 0.74- -0.37)	−0.41 (− 3.12 - -0.06)	0.792

Data are presented as n (%) or median (25th–75th percentiles). *Fisher information test or Mann-Whitney U test; *one missing value. ^a^Congenital Diaphragmatic Hernia Study Group. Congenital diaphragmatic hernia: defect size correlates with developmental defect. J Pediatr Surg. 2013;48:1177e82. *p*-values in bold are significant (< 0.05)

Figure 2 depicts pre and post-BD Rrs 8 (panel A), Xrs 8 (panel B), and AX (panel C) values in relation to age: differences between pre and post-BD values decreased when age increased.

**Fig. 2:**
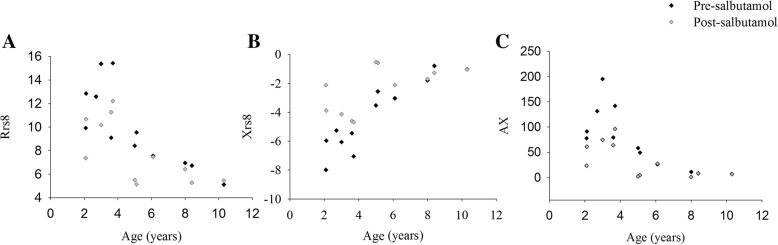
Distribution of pre and post BD Rrs8 (panel **a**), Xrs8 (panel **b**) and AX (panel **c**) and age. Black and grey squares represent the pre-BD and the post-BD measurements, respectively

This study reports the mid- and long-term consequences on respiratory impedance in a sample of children with non severe CDH after surgical repair. Significant differences between measured and predicted values for Rrs8, Xrs8 and AX were found, confirming that an impairment of respiratory resistance and reactance is detectable throughout childhood in CDH survivors. Moreover, according to previous studies, these data are suggestive of gradual normalization of lung function in CDH children over time [2, 9]. In this connection, we found that respiratory function was only impaired in children aged less than 5 years, whereas near normal respiratory impedance was observed in older children, probably thanks to compensatory lung growth. Abnormalities both in the resistive and in the reactance components of respiratory impedance might be due to the different degree of damage induced by prenatal CDH, resulting in a reduction in total number of alveoli and in hypoplastic lungs. Indeed, the observed significantly higher than predicted Rrs8 values account for an increased total resistance of the respiratory system. Furthermore, children with CDH showed an increased Xrs8 compared to predicted values, which probably has to be ascribed to heterogeneous development of the bronchial tree as well as peripheral obstruction and decreased lung compliance [10]. The finding of increased differences of all the measured FOT parameters compared to their predicted ones only in younger children suggests that lung hypoplasia could play the major role in impairment of lung function in children with non-severe CDH, at least until school age. Indeed, pathological examination demonstrated different degrees of lung hypoplasia with too few or to small alveoli and/or normal or diminished number of conducting airways, in CDH patients and in animal models [11]. Moreover, the abnormal lung tissue development could result in the formation of large-size alveoli or emphysema [12]. Data from animal models showed that in CDH fetuses, low frequency Rrs and Xrs are compromised, suggesting that lung hypoplasia most likely affects mechanical properties of peripheral lung tissue [11]. The lung tissue distortion may contribute to an increase in lung elastic recoil [13]. Thus, the impaired respiratory impedance resulting in more negative values of reactance, suggests that FOT is able to describe the increase in energy expenditure to overcome the elastic components of the lung. Overall, this changes may introduce gas distribution inhomogeneity, which could be properly investigated through the application of a more suitable test, such as the Multiple Breath Washout. Therefore, future studies could be useful to explore this research area and to examine possible correlations with FOT. With regard to bronchodilator response, we did not observe significant differences between the two age subgroups.

Our study faced certain limitations. Firstly, given the small sample size, the current results need to be confirmed in larger populations. Secondly, the present data cannot be generalized to children with more severe CDH as half of the children in our sample were affected by type “B” defect (small - < 50%- portion of the chest wall devoid of diaphragm tissue), 75% had primary closure and only an average 6-day artificial ventilation with no need of extracorporeal membrane oxygenation. Lastly, the cross-sectional design of the study is another potential weakness; a longitudinal follow-up evaluation would be useful in assessing the sequential changes in pulmonary function.

This is the first study in children with CDH evaluating both resistance and reactance components of respiratory impedance using FOT. This technique was able to detect alterations in the respiratory function in young children with CDH, shedding some light on a grey zone that cannot be thoroughly assessed by forced expiratory maneuver using spirometry. Indeed, FOT has proved to be more sensitive than forced expiration in investigating subclinical airway abnormalities [14] and is considered a useful tool for evaluating basal airway obstruction and its reversibility in young children [3, 15].

In conclusion, in patients with non-severe CDH who underwent surgical repair, an impairment of respiratory impedance persists until school age; additionally, the higher, although not significant, difference in both the resistance and reactance components after bronchodilator suggests that changes in airway tone can be easily detected by the application of FOT in these children. Despite the small sample size, the present results suggest that a multidisciplinary follow-up including the evaluation of respiratory impedance should be performed in children with non-severe CDH, at least until school age [16].

## Data Availability

The datasets used and/or analysed during the current study are available from the corresponding author on reasonable request.

## Abbreviations

AX: Area under the reactance curve
CDH: Congenital diaphragmatic hernia
FOT: Forced Oscillation Technique
Rrs: Resistance
Xrs: Reactance
